# Cannabis as a Source of Approved Drugs: A New Look at an Old Problem

**DOI:** 10.3390/molecules28237686

**Published:** 2023-11-21

**Authors:** Adi Gabarin, Ludmila Yarmolinsky, Arie Budovsky, Boris Khalfin, Shimon Ben-Shabat

**Affiliations:** 1 The Department of Clinical Biochemistry & Pharmacology, Faculty of Health Sciences, Ben Gurion University of the Negev, Beer Sheva 84105, Israel; adi.jbaren@gmail.com (A.G.); yludmila@post.bgu.ac.il (L.Y.); boriskh83@gmail.com (B.K.); 2 Research and Development Authority, Barzilai University Medical Center, Ashkelon 7830604, Israel; arieb@bmc.gov.il

**Keywords:** cannabis, drugs, cannabidiol, tetrahydrocannabinol

## Abstract

Cannabis plants have been used in medicine since ancient times. They are well known for their anti-diabetic, anti-inflammatory, neuroprotective, anti-cancer, anti-oxidative, anti-microbial, anti-viral, and anti-fungal activities. A growing body of evidence indicates that targeting the endocannabinoid system and various other receptors with cannabinoid compounds holds great promise for addressing multiple medical conditions. There are two distinct avenues in the development of cannabinoid-based drugs. The first involves creating treatments directly based on the components of the cannabis plant. The second involves a singular molecule strategy, in which specific phytocannabinoids or newly discovered cannabinoids with therapeutic promise are pinpointed and synthesized for future pharmaceutical development and validation. Although the therapeutic potential of cannabis is enormous, few cannabis-related approved drugs exist, and this avenue warrants further investigation. With this in mind, we review here the medicinal properties of cannabis, its phytochemicals, approved drugs of natural and synthetic origin, pitfalls on the way to the widespread clinical use of cannabis, and additional applications of cannabis-related products.

## 1. Introduction

Cannabis plants grow wild and are cultivated in many countries. Generally, three species are recognized in the *Cannabis* genus: *C. sativa*, *C. indica*, and *C. ruderalis*. Nevertheless, some experts debate whether the *Cannabis* genus contains one species (*Cannabis sativa* L.) or two or three species. Whatever the case regarding the classification, the medicinal applications of cannabis plants have been widely acknowledged since the Neolithic period, according to genetic, historical, and archaeological evidence [1,2,3]. Throughout its history, cannabis has been an important medicinal plant and has also been used as a fiber, food, and oil [4].

Despite the medicinal importance of the cannabis plant, it is important to note that according to the World Health Organization’s (WHO) estimates, cannabis is the most widely grown, trafficked, and abused illicit drug, consumed by around 147 million people [5]. In fact, marijuana is the most widespread illicit substance among adolescents and adults, sometimes surpassing tobacco use in many countries [6]. Marijuana use tends to steadily increase among teenagers [7]. Cannabis use during adolescence has been shown to be connected with poor cognitive function and psychological symptoms, such as psychosis, mania, and suicidality [8].

With regards to the effects of the cannabis plant on health and wellbeing, it is important to differentiate between marijuana and hemp. While the term marijuana is mainly used with regards to recreational applications of the plant [9,10], the term hemp is used with regards to medicinal compounds, fibers, and seeds [11]. The difference between these terms could be also a botanical one, as marijuana is used to denote the flowering tops, seeds, stems, and leaves from *C. sativa*, while hemp is defined as the fibrous component of the plant [12]. In addition, hemp is pharmacologically defined as a cannabis plant with a total delta-9 tetrahydrocannabinol (THC) concentration below 0.3% (*w*/*w*), while marijuana contains above 0.3% (*w*/*w*) THC [13].

Despite the potential dangers of cannabis use, it is clear that the therapeutic potential of cannabis plants is enormous. In fact, 565 unique compounds with medicinal potential have been identified in or isolated from *C. sativa* [14]. Among them, there are at least 120 phytocannabinoids and additional phytochemicals such as terpenes, polyphenols, fatty acids, different organic acids, simple ketones, simple esters/lactones, simple alcohols, alkaloids, and polysaccharides [15]. The medicinal properties of some of these compounds will be further described in the upcoming sections of this review.

Given that the medical and scientific communities have become increasingly interested in using cannabis and its phytochemicals for therapeutic purposes, the question is why so few cannabis-related approved drugs exist. Here, we will try to answer this important question. Additionally, we will describe the medicinal properties of cannabis, its phytochemicals, approved drugs of natural and synthetic origin, pitfalls on the way to the widespread clinical use of cannabis, and additional applications of cannabis-related products.

## 2. Medicinal Properties of Cannabis

### 2.1. General Overview of Medicinal Properties of Cannabis and Their Mode of Action

There are many scientific studies supporting the medicinal properties of cannabis, including its anti-inflammatory [16,17,18], anti-diabetic [19], neuroprotective [20,21], anti-cancer [22,23,24], anti-oxidative [25], anti-microbial [26,27,28], anti-viral [29], and anti-fungal activities [30]. Among the diseases, disorders, syndromes, and health conditions affected and treated by cannabis are epilepsy, Alzheimer’s disease, Parkinson’s disease, post-traumatic stress disorder (PTSD), skin diseases, and cancer or its subsequent side effects like appetite loss, chronic pain and nausea [31].

Different phytochemicals in cannabis contribute to its medicinal properties, depending on their concentrations, stability, volatility, pharmacological actions, physicochemical parameters, and combinations [32,33]. Several approaches exist for the description of cannabis chemotypes. For example, five chemotypes of cannabis have been reported based on their predominant terpenes: (i) β-myrcene, (ii) α- and β-pinene, (iii) β-caryophyllene and limonene, (iv) β-caryophyllene, and (v) terpinolene [34].

It is important to stress that active compounds in many plant extracts have synergistic or antagonistic effects on various activities [35]. Synergy is defined as a condition in which the effect of two or more compounds functioning in combination is greater than the expected additive effect of these compounds in separation [36]. Synergistic interactions between phytocannabinoids and other phytochemicals in cannabis allow it to have various therapeutic benefits. Many of them have not yet been investigated or have not been researched deeply. Sometimes, an antagonistic effect takes place rather than a traditional synergism. One interesting example of such an effect is the ability of two bicyclic monoterpenoids of cannabis (α-pinene and β-pinene) to inhibit acetylcholinesterase in the brain, allowing for memory improvements and decreases in the cognitive dysfunction caused by THC intoxication [37]. The synergistic mechanisms of action of phytocannabinoids and terpenoids in cannabis may make them effective in treating allodynia, itch, and other types of pain involving superficial sensory nerves and skin [38,39,40]. The following mechanisms of synergy are known [41]: multi-target effects; pharmacokinetic effects; and modification of adverse events. In addition, phytocannabinoids and terpenes in cannabis may be used to treat diabetes and its complications [42].

Interactions between the various phytochemicals in cannabis produce so-called entourage effects, whereby the active agent in the presence of its entourage compounds may have better therapeutic activity than the natural products on their own [43,44]. Although the entourage effect was first discovered by Ben-Shabat et al. in 1998 [43,45], many combinations of cannabis components have not been researched yet. Many experimental studies demonstrate an “entourage effect” that might be deduced from the medicinal properties of cannabis extracts relative to the singular molecules they contain [46]. Once the idea of the entourage effect is well understood, interactions between phytocannabinoids, terpenoids, and phenylpropanoids in cannabis may be defined as synergistic [44].

### 2.2. The Endocannabinoid System (ECS)

The endocannabinoid system (ECS), or endocannabinoidome, is defined as a multifunctional signaling structure of the mammalian nervous system and many other peripheral tissues [47]. The ECS regulates many important processes in the human body, including pain perception and transmission [48], gastrointestinal, hormonal, and cardiovascular activities, immune function, and inflammation reactions [49]. In addition, the ECS participates in food intake and energy metabolism, regulates the hypothalamic–pituitary–adrenal axis, and influences various emotional and behavioral conditions [50,51]. 

The ECS consists of cannabinoids, cannabinoid receptors, and the proteins that transport, synthesize, and degrade endocannabinoids. Cannabinoids are categorized into three groups: endogenous cannabinoids, phytocannabinoids, and synthetic cannabinoids [52]. Endocannabinoids are endogenous human lipids activating cannabinoid receptors [53]. Endocannabinoids are derivatives of polyunsaturated fatty acids and include N-arachidonoylethanolamide (anandamide, AEA), 2-arachidonoylglycerol (2-AG), 2-arachidonyl glyceryl ether (noladine, 2-AGE), virodamine (O-arachidonoyl ethanolamine), and N-arachidonoyl-dopamine (NADA) [52]. Endocannabinoids are essential regulators of synaptic function in the central nervous system and neural processes including cognition, motor control, pain, and feeding behavior [54,55].

CB1 and CB2 are the best-characterized cannabinoid receptors. CB1 receptors are primarily found in the central nervous system, adipose tissue, liver, pancreas, skin, and skeletal muscle [56,57]. CB2 receptors are found in macrophages, lymphocytes and natural killer cells, in neurons, and in some peripheral tissues [57]. Other receptors include the orphan GPCRs GPR55, GPR18, and GPR3–6–12 subsets, as well as ionotropic receptors. Transient receptor potential (TRP) channels are membrane proteins which participate in the process of the transduction of chemical and physical stimuli. It has been proposed that the downstream signaling of this system is mediated by TRP channels [57]. Peroxisome proliferator-activated receptors (PPARs) are nuclear receptors which regulate the expression of many target genes involved in various processes [58]. Both are G protein-coupled receptors (GPCRs), primarily coupling to inhibitory G proteins. They inhibit adenylyl cyclase and specific voltage-sensitive calcium channels, stimulate mitogen-activated protein kinases (MAP kinases), inwardly rectify potassium channels (GIRKs), and recruit beta-arrestins, among other actions [59].

**Figure 1:**
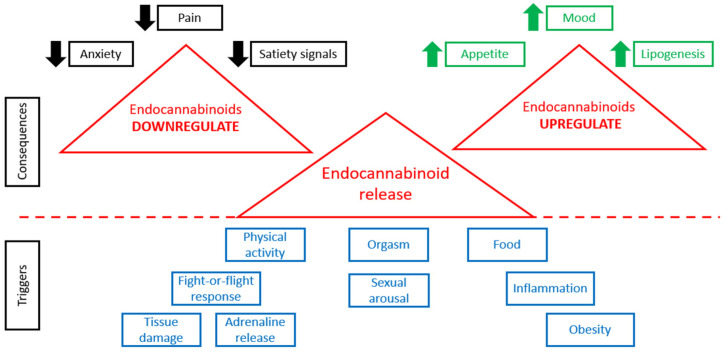
ECS in normal and pathological conditions.

When physiological conditions are normal, the tonic activity of the ECS is minimal [60]. Any change in ECS tone (increase or decrease) is associated with various pathological states [61]. Figure 1 demonstrates that the release of endocannabinoids is triggered by various stimuli, such as physical activity, various stresses, food consumption, sexual behavior, orgasm, obesity, inflammation, tissue damage, and so on. Higher levels of endocannabinoids are connected with many consequences (Figure 1). In case of alterations in the concentrations of endogenous endocannabinoid ligands, receptor expression or activity, or endocannabinoid metabolic enzyme activity, various pathological conditions are observed [62].

### 2.3. Neurological Disorders

The ECS plays a key role in the pathogenesis of many neurological diseases, including multiple sclerosis (MS), epilepsy, Alzheimer’s disease (AD), Parkinson’s disease (PD), and Huntington’s disease (HD) [51]. In fact, the majority of clinical studies with regards to the potential benefits of cannabis are associated with diseases of the central nervous system [63].

#### 2.3.1. Multiple Sclerosis (MS)

While there is no cure for MS, cannabis has proven helpful. There are some medications for reducing symptoms of spasticity and MS-related pain, including nabiximols (Sativex oral spray), oral cannabis extract, and synthetic THC [64].

#### 2.3.2. Alzheimer’s Disease (AD)

It has been reported that cannabis significantly reduces the brain inflammation implicated in AD [65]. In vivo experiments using AD models showed that low-dose synthetic cannabinoids administered for a short time (WIN 55,212-2 0.01 mg, 7 days) prevented inflammatory responses [66]; low-dose use of synthetic cannabinoids for a more extended period (JWH-133 0.2 mg/kg, 4 months) was effective in decreasing inflammation [67]; administration of THC and cannabidiol (CBD) together helped to preserve memory; and a single use of an ultra-low dose (0.002 mg/kg) of THC prevented inflammatory responses [68]. It is known that brain inflammation and oxidative stress are fundamental mechanisms causing AD; therefore, the above-mentioned results could be explained and attributed to the anti-oxidative properties of cannabinoids [69,70]. There is little information on the clinical effects of cannabinoids in patients with AD. Some reports have demonstrated, on the one hand, negative influences of cannabis on motor inhibition, attention, and episodic control, while, on the other hand, beneficial effects on some dementia-related symptoms have been observed [71,72]. Data derived from THC and nabilone tests for treating AD showed neuropsychiatric symptoms did not change after treatment; nevertheless, some beneficial effects on balance and gait were reported [71,72,73].

#### 2.3.3. Parkinson’s Disease (PD)

Although, according to clinical studies, there is no scientific evidence recommending the use of cannabis in PD patients, some studies showed tremor, anxiety, and pain were decreased and sleep quality was improved in these patients following cannabis use [51]. With regards to post-traumatic stress disorder, some clinical studies reported a decrease in stress and anxiety symptoms after cannabis treatment [65,72], but they were performed using relatively small samples.

#### 2.3.4. Chronic Neuropathic Pain

Chronic neuropathic pain is a pathological condition that is very difficult to treat successfully. Clinical studies demonstrated that cannabis products (inhaled herbal cannabis, sprays or tablets containing natural active cannabis compounds or synthetically obtained agents) have significant analgesic properties [74]. To the best of our knowledge, there have been no longitudinal clinical studies examining the effects of cannabis use on pain-related outcomes over a period of time.

It has been reported that when THC or CBD was administered as adjunctive therapy to other drugs and psychotherapy, an improvement was observed in the condition of patients with dementia, schizophrenia, cannabis and opioid dependence, general social anxiety, anorexia nervosa, post-traumatic stress disorder, and Tourette`s disorder [64,65,66,72,73,74,75].

### 2.4. Skin Diseases

Interestingly, human skin also participates in the ECS, and there are endogenous ligands in the skin that interact with two primary cannabinoid receptors [75]. However, these receptors exist in many skin structures, including epidermal keratinocytes, melanocytes, dermal cells, mast cells, sweat glands, hair follicles, and cutaneous nerve fibers [76,77]. The skin’s ECS takes part in many important cellular processes, including the proliferation, differentiation, apoptosis, and regulation of inflammatory and immune responses [78].

Although there are no market-approved drugs based on cannabis for the treatment of skin diseases, it has been found that cannabinoids are effective in the treatment of many dermatological pathologies, such as acne vulgaris, allergic contact dermatitis, eczema, pruritus, psoriasis, and skin cancer [77,79]. It has been reported that cannabinoids decrease keratinocyte proliferation, which could be the reason for their effectiveness in treating psoriasis [80]. CBD and cannabigerol were also effective in the treatment of psoriasis [81].

#### Skin Wound Healing

Extract of cannabis improves skin wound healing by decreasing inflammation. Specifically, in human keratinocytes and fibroblasts, such an effect is associated with the inhibition of nuclear factor-kappa B activity [82]. It has also been reported that cannabis extract and cannabidiol decreased TNF-α and IL-1β production in LPS-induced RAW 264.7 cells and enhanced wound healing in human gingival fibroblasts [83]. In addition, wound healing was enhanced because of improved collagen production [82].

It is important to note that infection, inflammation, and other skin diseases significantly inhibit wound healing. Hexane extracts from cannabis seeds are rich in polyunsaturated fatty acids with anti-inflammatory and anti-bacterial properties [84]. It was demonstrated in a cell model that these extracts had anti-microbial action against *Propionibacterium* and decreased inflammatory markers in keratinocytes [85]. In another clinical study, 3% cannabis seed extract cream was applied twice a day for 12 weeks on the right cheek of 11 patients with sebum and erythema (the left cheek was a control). A significant reduction in sebum level was reported [86].

## 3. Cannabinoids in Light of Current Knowledge (Approved Drugs)

### 3.1. Approved Drugs of Natural Origin

We have mentioned that phytocannabinoids are the most active compounds in *C. sativa*; they contain an isoprenyl residue, a resorcinyl core, and a side chain [87], and they also have the typical C21 terpenophenolic skeleton [14]. The most widespread compounds are THC and CBD. CBD has exhibited various pharmacological activities in many preclinical and clinical studies. It can treat inflammation, cancer, cardiovascular diseases, epilepsy, and neurodegenerative and psychiatric disorders [24,88,89,90,91,92,93,94,95,96]. Its therapeutic properties are well-known [97]. Δ^9^-THC is the main active constituent of cannabis, with many medicinal properties, such as anti-emetic and analgesic (especially helpful for cancer patients), anti-anxiety, anti-inflammatory, and so on [98]. Other important natural cannabinoids are cannabinol (CBN), cannabichromene (CBC), cannabigerol (CBG), cannabicyclol (CBL), and tetrahydrocannabivarin (THCV) [99].

**Figure 2:**
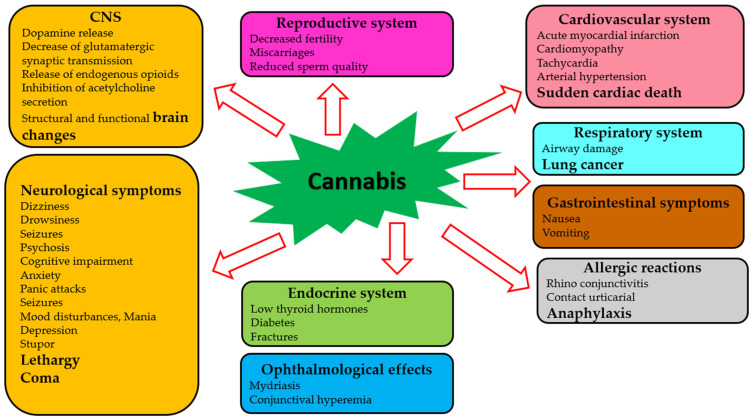
Adverse effects of cannabis on human systems.

Although clinicians, pharmacologists, and researchers strive to reach the therapeutic potential of cannabis to its fullest extent, there are many challenges associated with drug trials and approvals. A major problem is that different countries have different laws regarding the use of cannabis for medical and recreational purposes [100,101,102]. Considering the complexity and pleiotropy of cannabis’ actions, a comprehensive understanding of the molecular pharmacology of its components is necessary. The effects of cannabis and its phytochemicals depend on many factors, including pharmacological targets, drug preparation, concentration, and the chosen route of administration [103]. In addition, adverse effects of cannabis on almost all body systems have been reported [104,105,106]. Several factors determine the severity of these adverse effects, including genetic variation, age, sex, ethnicity, and the concentration of the active agent [107,108,109]. Table 1 and Figure 2 summarize the adverse effects of cannabis.

The majority of publications devoted to the harmful effects of cannabis focus on the central nervous system [110,111]. A single exposure to cannabinoid agonists may result in a sequence of biochemical changes in the brain, including an increase in dopamine release, a decrease in glutamatergic synaptic transmission, release of endogenous opioids, and an inhibition of acetylcholine release [112]. Among heavy cannabis users, significant changes have been observed in the brain’s structure and function [113,114]. Some severe neurological symptoms (dizziness, drowsiness, seizures, coma, and others) may occur [115,116,117]. The risk of psychotic conditions increases when cannabis is consumed acutely and repeatedly [118,119]. Some cognitive impairments occurring during acute cannabis intoxication have been reported [120]. Anxiety and panic attacks have also occurred in naive users of cannabis [121]. Furthermore, chronic use of cannabis can lead to mood disturbances, mania, and depression [122,123]. It was found that neurological symptoms such as stupor, lethargy, seizures, and even coma have occurred in children due to cannabis toxicity [124,125].

Adverse ophthalmological effects (mydriasis and conjunctival hyperemia) have been reported [124]. Cannabis use has been suggested to be linked to acute myocardial infarction, cardiomyopathy, and sudden cardiac death [126]. Additional effects of cannabis on the cardiovascular system are related to tachycardia and arterial hypertension [127]. Respiratory effects of cannabis have been studied in cases of acute cannabis consumption, and decreased airway resistance has been reported [128,129]. Chronic cannabis use has been linked to an increased risk for developing airway diseases and lung cancer [130,131]. Several studies have demonstrated gastrointestinal symptoms (nausea, vomiting, and thirst) after using cannabis [124,132]. Decreased fertility, miscarriages, reduced sperm function, lowering of thyroid hormones, and increased risk of diabetes and fractures can all be caused by the effects of cannabis and cannabinoids on the endocrine system [133]. Some compounds of cannabis can trigger various allergic reactions, such as rhinoconjunctivitis, contact urticaria, and anaphylaxis [134].

**Table 1:** Adverse effects of cannabis intake on various human systems.

Human System	Adverse Effects	References
Central nervous system	Biochemical and structural changes in the brain Some severe neurological symptoms	[112,113,114] [115,116,117]
Reproductive system	Decreased fertility, miscarriages, reduced sperm function	[133]
Cardiovascular system	Tachycardia and arterial hypertension	[127]
Respiratory system	Decreased airway resistance	[128,129,130,131]
Gastrointestinal system	Nausea, vomiting, thirst	[124,132]
Endocrine system	Lowering of thyroid hormones, increased risk of diabetes	[133]

Clinical complications may occur due to the interactions between approved drugs and cannabis compounds [135]. Sometimes, these interactions may lead to enhanced drug responses and modified or unexpected adverse reactions [136]. In vitro and in vivo studies have shown that cannabinoids can affect the metabolism of various drugs by acting on P450 isoenzymes [137]. These additive effects may result in tachycardia, hypo-, or hypertension. Administration of cannabinoids with CNS depressants, such as alcohol, muscle relaxants, opioids, and anti-cholinergics, can also cause tachycardia and confusion [138]. There have also been studies and case reports indicating potential drug interactions with warfarin, oxymorphone, pentobarbital, cocaine, sympathomimetic amines, disulfiram, disulfiram, etc. [139,140], but further research is needed.

Thus, the results of many preclinical studies and clinical data are inconclusive about the overall usefulness of cannabis and its compounds for treating many diseases. Interestingly, drugs based on plant material made up more than one-fifth (22%) of all NME (new molecular entity) drugs approved before 1950, but have declined by more than 50% to 8.7% since that time [141].

Cannabis, cannabis-based medicine, and phytogenic or synthetic cannabinoids could be administered by various routes, such as smoking, vaporization, oral, oro-mucosal, and other routes, which affect the absorption and toxicity of cannabinoids [142].

The oral administration route of cannabinoids has many advantages as it ensures safety, excellent patient compliance, ease of ingestion, pain avoidance, and the possibility of accommodating various types of drugs [143]. Obtaining suitable formulations for oral administration is the most challenging process due to the lipophilic nature of cannabinoids, so there are some approaches to improve oral bioavailability. One of them is the administration of cannabinoids with lipids [144]. Some nanoparticle-based formulations have been created [145,146]. Synthetic nanomicellar cannabinoid formulations have improved cannabinoids’ oral bioavailability [147]. In addition, some nanoemulsions that contain cannabinoids have been developed [148,149].

Throughout the procedure of drug development, many obstacles need to be overcome. Only if all preclinical and clinical studies demonstrate that a drug’s potential therapeutic benefit outweighs its side effects, toxicity, and so on, that its chemical content and manufacturing information is perfect, and that it is possible to receive financial assistance from sponsors, foundations, and private companies, will a new drug submission be filed [150]. For example, nabiximol, which has similar percentages of two components, cannabidiol and delta-9-tetrahydrocannabinol, has been approved by regulatory authorities in Canada and in many European countries for the treatment of MS-related neuropathic pain and spasticity [151].

For cannabis to enter clinical practice, its market application has to be approved by the Food and Drug Administration (FDA). Table 2 contains precise information about all approved cannabis-related drugs. So far, the FDA has approved one cannabis-derived drug and three synthetic cannabis-related drug products (Table 2). It is important to note that nabilone (Cesamet; Valeant Pharmaceuticals North America) and dronabinol (Marinol; Solvay Pharmaceuticals) are synthetic analogs of THC [152]. Sativex (GW Pharmaceuticals, UK) is a mixture of two cannabis extracts enriched with THC and CBD; their approximate ratio is 1:1. This drug was licensed in Canada in 2005 [153].

An online survey on the use of cannabis in patients suffering from amyotrophic lateral sclerosis demonstrated that 21.7% of respondents had improvements in motor (rigidity, cramps, fasciculation) and non-motor (sleep quality, pain, emotional state, quality of life, depression) symptoms and only 6.2% noted drowsiness, euphoria, and dry mouth [154].

### 3.2. Synthetic Cannabinoids—Phytocannabinoids Derivatives

More than 450 synthetic cannabinoids have been developed since the second half of the twentieth century; they have some structural similarities to phyto- and endocannabinoids [155,156]. There are several classifications of synthetic cannabinoids. One of them focuses on the following structural groups: adamantoylindoles, aminoalkylindoles, benzoylindoles, cyclohexylphenols, dibenzopyrans, naphthoylindoles, naphthylmethylindoles, naphthylmethylindenes, naphthoylpyrroles, phenylacetylindoles, tetramethylcyclopropyl ketone indoles, quinolinyl ester indoles, and indazole carboxamide compounds [157]. It has also been reported that chemical synthesis could be a source of bioactive compounds of different structures, such as classical cannabinoids, non-classical cannabinoids, hybrid cannabinoids, aminoalkylindoles, eicosanoids, and miscellaneous cannabinoids [158].

Although synthetic cannabinoids can produce desired effects, such as relaxation, euphoria, and disinhibition, similar to those of THC, they give rise to many serious or even fatal adverse events [155,159]. The toxicological effects of synthetic cannabinoids are summarized in Table 3. Synthetic cannabinoids can bind to CB1 and CB2 receptors with higher efficacy than THC [160,161]. The mechanism of action of synthetic cannabinoids is associated with enhanced signaling of cannabinoid receptors or the disruption of mitochondrial homeostasis [162]. Nevertheless, the mode of action of synthetic cannabinoids has been poorly investigated.

## 4. Additional Applications of Cannabis

### 4.1. Unregulated Cannabis Products

#### 4.1.1. Cannabis Products for Topical Treatment

Cannabis products designed for topical treatment have gained significant popularity due to their potential therapeutic benefits. These products may be in the form of creams, balms, lotions, or oils infused with cannabinoids like CBD or THC. When applied to the skin, they provide localized relief from pain, inflammation, arthritis, psoriasis, and other skin disorders. Unlike ingested forms of cannabis, topical treatments do not produce psychoactive effects because they typically do not penetrate the epidermis into the bloodstream [171]. It makes them an attractive option for individuals seeking the potential therapeutic advantages of cannabis without the associated high risks. However, it is recommended to carefully research these products and choose reputable ones, as the quality, potency, and safety of cannabis-infused topicals can vary widely in an unregulated market [171]. Moreover, unregulated cannabis products for topical treatment raise a serious concern within the burgeoning cannabis industry. The lack of stringent oversight and quality control measures can lead to inconsistencies in potency, safety, and efficacy. Without proper regulation, there is limited accountability for producers and distributors, making it challenging to address any adverse effects or ensure product consistency. To ensure the safety and effectiveness of cannabis-infused topical treatments, comprehensive regulatory frameworks are essential to provide consumers with reliable, standardized products that can deliver therapeutic benefits while mitigating potential risks.

#### 4.1.2. Cannabis Products for Systemic Treatment

Cannabis products designed for systemic treatment have gained considerable attention for their potential therapeutic applications. These products typically include edibles, tinctures, capsules, or oils that contain cannabinoids like CBD and THC. When ingested, they are absorbed into the bloodstream, allowing for a more widespread and prolonged effect throughout the body. Systemic cannabis products are being explored for various medical conditions, including chronic pain, epilepsy, anxiety, and nausea associated with chemotherapy. However, their administration requires consultation with healthcare professionals and adherence to the prescribed dosages, as the psychoactive effects of THC could be significant and vary from person to person. As regulatory frameworks evolve, the availability and safety of systemic cannabis products are expected to improve, offering potential relief to patients seeking alternative treatments for their ailments [172,173,174].

Unregulated cannabis products for systemic treatment present a concerning aspect of the cannabis industry. The lack of comprehensive oversight and quality control measures can lead to significant risks for consumers. Ingestible cannabis products, such as edibles and tinctures, may contain inconsistent levels of cannabinoids like THC and CBD, posing challenges for proper dosing and potentially causing adverse effects. The absence of rigorous testing and labeling requirements also leaves room for contamination by harmful substances, making these unregulated products a potential health hazard. To ensure the safety and efficacy of cannabis-based treatments for systemic use, robust regulations and standards are imperative, allowing patients to access reliable, standardized products while minimizing the risks associated with the consumption of unregulated cannabis.

#### 4.1.3. Food Additives

Cannabis additives in food are not frequently consumed products in the human diet. Although cannabis seeds, roots, and flowers have a high nutritional value [175], they are not a part of basic human nutrition. The seeds of cannabis are rich in digestible proteins, lipids, PUFAs, insoluble fiber, carbohydrates, natural anti-oxidants, and bioactive components such as phenolic compounds, bioactive peptides, carotenoids, tocopherols, and phytosterols, and they have a healthy balance between omega-3 and omega-6 fatty acids [176]. In addition, its proteins have the highest biological value because of the sulfur-containing amino acids methionine, cystine, and arginine, which are essential amino acids with beneficial cardiovascular properties [177]. 

It is well known that active compounds in food may enhance physiological activity and promote human health. Cannabis has a high content of valuable phytochemicals and as such may be used for various food preparations such as oils, oil-filled capsules, or tinctures in medicinal practice. Seeds are primarily compressed to extract oil. Seeds could also be added to several other products such as yogurts, hemp flour, baked products, hemp milk, pralines, chocolates, and so on [178]. Cannabis additives in yogurt enhance its nutritional properties, including protein content [179]. The addition of hemp flour to cookies, bread, and pasta results in enhanced total phenolic content, anti-oxidant activity, ash, protein, and fat contents [180,181,182]. The principal components used in the preparation of cannabis edibles are oil and butter [183,184,185]. In addition, its sprouts, leaves, and flowers are edible in juices or salads; they have many polyphenols and cannabinoids that seeds do not contain or contain in trace amounts [186,187]. When cannabis extract serves as an additive to chocolate, chocolate’s physical and nutritional properties improve significantly without changing its qualities [188]. It has been reported that the incorporation of cannabis into beer makes the beverage more “elevated” and “relaxing” [189]. Altogether, the pressurized liquid extraction, solid-phase extraction, matrix solid-phase dispersion, and microwave-assisted extraction of cannabis are frequently used in the food industry [190].

There is interest in cannabis (hemp) milk because it could serve as a possible alternative to cow’s milk. Its disadvantages are the low content of certain minerals, for example, iron, vitamins, and amino acids, the possibility of milk allergy, lactose intolerance, and hypercholesterolemia. Nevertheless, cannabis (hemp) milk could be a good substitute for cow’s milk for vegetarians and vegans. It can easily be made at home using water and hemp seeds. This beverage is a source of high-quality plant protein, good fats, and essential minerals. It has been demonstrated to have anti-thrombotic, anti-vasoconstrictive, anti-inflammatory, and neuroprotective properties, and also to reduce vomiting [191]. Hemp milk is also suitable for smoothies, coffee, cereal, cappuccinos, lattes, and so on [175].

Cannabis extract has significant anti-microbial activity against foodborne pathogens in meat [192]. Cannabis extract and its components have great potential as a natural preservatives for food industry because the most common food preservatives, such as sodium benzoate, acetic, lactic, benzoic and sorbic acids, or hydrogen peroxide have many adverse health effects [35].

Due to its high potential, there is a projection that the market for cannabis foods will expand by USD 22.18 billion during 2020–2024 [178]. As a product with huge market potential, it is imperative to develop advanced analysis and processing technologies for cannabis-based food additives. Targeted delivery technologies from the field of pharmacology may be used in food processing, which will help to maximize the medicinal properties of cannabis extracts and improve their bioavailability. Future research should focus on flavor and taste, sensory differences among different formulations, and consumers’ preferences.

### 4.2. Alternative Medicine

Often, complementary and alternative treatment with cannabis can step in when the patient would like a complementary approach or when conventional treatments have not proven to be effective. Complementary and alternative medicine uses may include non-standardized cannabis products with unknown amounts of THC and CBD [137]. Such treatments are commonly applied in patients with inflammatory bowel disease (ulcerative colitis and Crohn’s disease) [193]. About 10–15% of treated patients had an improved appetite and relief of some symptoms such as nausea, diarrhea, and abdominal pain [194,195]. Another study showed that in almost one-third of patients, there was a better relief of the above-mentioned symptoms as compared to steroid treatment [196].

Complementary and alternative treatment with cannabis has been also evaluated in patients with amyotrophic lateral sclerosis; cannabis moderately reduced symptoms of pain, spasticity, drooling, appetite loss, and depression in these patients [197]. Unfortunately, in the case of Parkinson’s disease and cancer, it was not possible to estimate the effect of alternative treatment with cannabis due to small sample sizes [198,199]. On the other hand, complementary and alternative treatment with cannabis oil was effective in patients with refractory epilepsies, nausea and vomiting caused by chemotherapy, and chronic and neuropathic pain associated with spasms [200].

Although CBD oil-containing products are sold in many health food stores and drugstores in the form of food supplements, toothpastes, mouth sprays, and drops, their effect on the oral cavity has not been investigated yet [201]. Cannabis-based lotions, creams, ointments, oils, and salves are produced by the cosmetic industry [202]. Since we have previously mentioned the anti-microbial properties of cannabis [26,27,28], it seems plausible that its extracts and phytochemicals may be used as cosmetic preservatives.

## 5. Pitfalls on the Way to Widespread Clinical Use of Cannabis and Cannabis-Derived Compounds

The path to the widespread clinical use of cannabis faces several potential pitfalls. These pitfalls include the following:

Limited research and clinical trials: Despite growing interest, there is still a limited body of rigorous clinical research on the therapeutic effects of cannabis. Research exploring the potential of cannabis for treating various medical conditions is constrained by a scarcity of studies, which frequently fall short in terms of the scientific rigor, controls, and sample sizes necessary for drawing meaningful clinical results. This lack of comprehensive scientific data makes it challenging for healthcare professionals to confidently recommend cannabis-based treatments [203];

Regulatory hurdles: The FDA still classifies cannabis as a Schedule I drug, which means it has no accepted medical use and a high potential for abuse. This classification makes it difficult to conduct research and hinders the development of standardized, pharmaceutical-grade cannabis products [204];

Dose standardization and quality control: Ensuring consistent dosage and quality of cannabis products is crucial for clinical use. Variabilities in potency and composition among different strains and products can lead to unpredictable effects and makes it hard to establish standardized treatment protocols. The use of a standard unit dose of cannabis and cannabis-derived compounds in research is an important step for improving our ability to understand both the adverse effects of cannabis and the drug’s medical potential [205];

Adverse effects and safety concerns: Cannabis has side effects, particularly in higher doses or when used by certain populations (e.g., adolescents, pregnant individuals, people with certain mental health conditions, and so on) [206]. Consistent patterns have surfaced from a range of influential papers and an increasing body of recent research, presenting substantial proof that exposure to cannabis during the prenatal, perinatal, and adolescent stages can lead to a varied range of cognitive and behavioral changes in adulthood. This is achieved by disrupting multiple neurobiological systems within the brain regions associated with psychotic and affective disorders. Whether this risk ultimately leads to psychiatric and substance use disorders hinges on a variety of factors, including genetics, sex, and environmental circumstances, which will be more comprehensively elucidated as research in this domain progresses [207];

Interactions with other medications: Cannabis has the potential to interact with other medications, potentially impacting their effectiveness or leading to undesirable side effects. Numerous studies have shown that primary cannabinoids and their relevant metabolites found in the plasma of cannabis users can inhibit various P450 enzymes, such as CYP2B6, CYP2C9, and CYP2D6 [208]. These findings imply that circulating cannabinoid metabolites play a pivotal role in the inhibition of CYP450 enzymes, which can have implications for potential drug interactions. This is particularly critical for patients who are using multiple medications [209];

Cannabinoid dual agonists: Despite its potential benefits in disease treatment, the use of THC is particularly hindered by the fact that it acts as an agonist on both CB1 and CB2 receptors, leading to psychotropic side effects associated with CB1 receptor activation. This limitation has restricted the clinical application of cannabinoid agonists. Consequently, there is a pressing need to develop synthetic cannabinoid analogs that can deliver the therapeutic effects of cannabinoids without causing undesired psychoactive properties. Intensive research in this direction is presently in progress, aiming to overcome the obstacles on the path to widespread clinical use [210];

Stigma and cultural attitudes: The acceptance of cannabis in clinical settings can be impeded by stigma and cultural attitudes. The misconceptions and historical stigma surrounding cannabis may hinder its recognition as a valid medical treatment. Numerous studies have emphasized that patients treated with cannabis-based medicinal products often face significant levels of perceived stigma from various segments of society. It is imperative for future research to delve into strategies aimed at diminishing this stigma both at an individual and community-wide level, with the goal of preventing discrimination against patients and likely enhancing their access to appropriate care [211];

Insurance and reimbursement issues: In many countries, medical cannabis treatment lacks insurance coverage, rendering it financially out of reach for numerous patients. In the absence of health insurance support, patients are required to personally cover the expenses, which can extend to hundreds of dollars each month. The elevated cost of medical cannabis products poses a substantial obstacle to its broader clinical adoption [212];

Ethical considerations: The increasing use of medical cannabis in the past decade raises several ethical considerations for the clinician. Regulatory challenges arise from disparities between the registration and certification of medical cannabis in different countries. Professional concerns stem from an inadequate understanding of the properties of cannabis and from the complex interplay between the physician, the patient, and commercial interests. Lastly, there are notable medical and psychological ramifications associated with the implementation of treatment plans. These ethical issues constitute significant pitfalls on the way to achieving a widespread clinical adoption of cannabis and cannabis-derived compounds [213].

To conclude, addressing these pitfalls requires concerted efforts from the medical and scientific communities, policymakers, and industry stakeholders. Rigorous research, clear regulations, standardized products, and education for healthcare professionals are essential steps towards achieving a wider clinical use of cannabis.

## 6. Conclusions

In this review, we present a large amount of solid evidence in support of the notion that cannabis extracts, phytocannabinoids, and other secondary cannabis metabolites have promising therapeutic potential, especially as analgesics, anti-emetics, and anti-inflammatory and neuroprotective agents. In fact, further research on the mechanisms by which secondary metabolites produce specific biological effects and how these molecules interact is warranted. Only knowledge of the modes of action of phytocannabinoids and other secondary metabolites will allow for the development of successful target-specific drug delivery systems. 

Serious difficulties and restrictions for research existed in many countries before the legalization of cannabis for medicinal use and recreational purposes. Although more than 37,000 publications devoted to cannabis have been known since 1841, the current literature base is insufficient to obtain comprehensive information about optimal product formulations and clinical trials. We hope that future clinically relevant studies will focus on improving the delivery of cannabis-based products.

## Figures and Tables

**Table 2:** Approved cannabis-related drugs.

Drug	Active Ingredient	Approved Use	Other Uses
Cannabidiol (Epidiolex^®^)	CBD	Rare, severe forms of epilepsy	Anxiety, anti-psychotic effects on schizophrenia, pain, a muscle disorder called dystonia, Parkinson’s disease, cancer, Crohn’s disease, and many other conditions
Dronabinol (Marinol^®^)	Synthetic cannabinoid: (6aR,10aR)-6a,7,8,10a-Tetrahydro-6,6,9trimethyl-3-pentyl-6H-dibenzo[b,d]-pyran-1-ol.	Anorexia associated with weight loss in patients with acquired immune deficiency syndrome. Nausea and vomiting associated with cancer chemotherapy	Stimulation of appetite, anti-emetic, analgesic, anti-cancer use, treating cannabis addiction, multiple sclerosis
Nabilone (CesametTM)	Synthetic cannabinoid: (±)-trans-3-(l,l-dimethylheptyl)6,6a,7,8,10,10a-hexahydro-l-hydroxy-6-6-dimethyl-9H-dibenzo[b,d]pyran-9-one	Nausea and vomiting associated with cancer chemotherapy	Fibromyalgia and multiple sclerosis; nightmares in post-traumatic stress disorder
Rimonabant (Acomplia^®^)	Rimonabant	Anti-obesity drug	Diabetes, drug dependence, cancer, atherosclerosis, smoking cessation
Nabiximols (Sativex^®^)	THC and CBD	Neuropathic pain, spasticity, overactive bladder, and other symptoms of multiple sclerosis	

**Table 3:** Toxicological effects of synthetic cannabinoids.

Toxicological Effects	Notes	References
Renal injury	Acute tubular necrosis	[163]
Cannabinoid hyperemesis syndrome	Nausea and vomiting	[164]
Cardiovascular effects	Acute myocardial infarction	[165]
Respiratory depression	Acute respiratory distress, pulmonary embolism	[166,167]
Effects on brain	Deficits in short-term memory, stroke, seizures, agitation, delirium, and psychosis	[168,169,170]

## Data Availability

Data are contained within the article.
